# Structural changes in the oral microbiome of the adolescent patients with moderate or severe dental fluorosis

**DOI:** 10.1038/s41598-021-82709-z

**Published:** 2021-02-03

**Authors:** Qian Wang, Xuelan Chen, Huan Hu, Xiaoyuan Wei, Xiaofan Wang, Zehui Peng, Rui Ma, Qian Zhao, Jiangchao Zhao, Jianguo Liu, Feilong Deng

**Affiliations:** 1 Special Key Laboratory of Oral Disease Research, Life Sciences Institute, Zunyi Medical University, Zunyi, China; 2 Special Key Laboratory of Microbial Resources and Drug Development, Life Sciences Institute, Zunyi Medical University, Zunyi, China; 3 Department of Animal Science, Division of Agriculture, University of Arkansas, Fayetteville, AR USA; 4 School of Stomatology, Zunyi Medical University, Zunyi, China

**Keywords:** Microbial ecology, Symbiosis

## Abstract

Dental fluorosis is a very prevalent endemic disease. Although oral microbiome has been reported to correlate with different oral diseases, there appears to be an absence of research recognizing any relationship between the severity of dental fluorosis and the oral microbiome. To this end, we investigated the changes in oral microbial community structure and identified bacterial species associated with moderate and severe dental fluorosis. Salivary samples of 42 individuals, assigned into Healthy (N = 9), Mild (N = 14) and Moderate/Severe (M&S, N = 19), were investigated using the V4 region of 16S rRNA gene. The oral microbial community structure based on Bray Curtis and Weighted Unifrac were significantly changed in the M&S group compared with both of Healthy and Mild. As the predominant phyla, Firmicutes and Bacteroidetes showed variation in the relative abundance among groups. The Firmicutes/Bacteroidetes (F/B) ratio was significantly higher in the M&S group. LEfSe analysis was used to identify differentially represented taxa at the species level. Several genera such as *Streptococcus mitis, Gemella parahaemolysans, Lactococcus lactis,* and *Fusobacterium nucleatum*, were significantly more abundant in patients with moderate/severe dental fluorosis, while *Prevotella melaninogenica* and *Schaalia odontolytica* were enriched in the Healthy group. In conclusion, our study indicates oral microbiome shift in patients with moderate/severe dental fluorosis. We identified several differentially represented bacterial species enriched in moderate and severe fluorosis. Findings from this study suggests that the roles of these bacteria in oral health and related diseases warrant more consideration in patients with moderate and severe fluorosis.

## Introduction

Dental fluorosis is endemic among certain areas in some countries, such as some economically underdeveloped areas in China^1^ and India^2^. It’s caused by excessive fluoride exposure over a long period during tooth formation and dental development, and therefore presents during childhood. The clinical characteristics of dental fluorosis are discoloration of the teeth (white specks to dark brown stains) and rough surface irregularities including pitting of the enamel. This damage is irreversible and therefore would have continuous influence on teeth aesthetics and oral health causing a profound negative impact on the quality of life^3^. Previous studies suggested that dental fluorosis was associated with several oral diseases, such as periodontitis^4^, and dental caries^5^. In a recent publication, Menya and colleagues found that moderate and severe dental fluorosis may increase the risk of esophageal squamous cell carcinoma (ESCC) in Africa^6^. Although there is a small amount of evidence to support the association between dental fluorosis and health issues, how the dental fluorosis make impact on the health of patients are poorly understood.

The oral cavity, one of the most diverse microbial habitat of the human body, is colonized by approximately 700 bacterial species^7^. Among these species are certain commensal microbiota that are partially responsible for various oral diseases. For example, *Streptococcus mutans*, the first isolated cariogenic bacteria, contributes considerably to dental caries formation^8^. Several other species, such as *Porphyromonas gingivalis, Bacteroides forsythus*, *Actinobacillus actinomycetemcomit*ans, and *Tannerella forsythia* are considered as periodontal pathogens^9^. A variety of periodontal pathogens are strongly implicated as risk factors in oral squamous cell carcinoma (OSCC)^10^. In addition, oral microbiota can cause a number of diseases directly (e.g. a number of systemic diseases via ectopic colonization) or indirectly (e.g. pancreatic cancer^11,12^, colorectal cancer^13^, and intestinal disease^14^). Although more evidence is needed to support and interpret the interrelationship between the oral microbiome and human health status, there is no doubt that it is important to control excessive fluoride exposure upon tooth enamel and to maintain good oral hygiene to minimize the damage from oral microorganisms.

The species composition of the oral microbiome is stable and yet susceptible to certain influences such as environment, host genetics, and health status of the host. Patients with dental fluorosis, especially severe dental fluorosis, find it more difficult to maintain oral hygiene by routine cleaning measures. Vora’s study^4^ revealed that there was a significant connection between severity of dental fluorosis and status of oral health. Thus, we hypothesized that dental fluorosis alters the oral microbiome. However, there appears to be an absence of research explaining how the oral microbiota change with the severity of dental fluorosis. The objective of the study was to investigate the changes of oral microbiota composition in different dental fluorosis states. In the present study, middle school students with similar life environment, nutrition, and age were recruited from Qinglong, Guizhou province, southwest China, an area where there is endemic coal-fired-pollution-induced dental fluorosis. We conducted the first-ever investigation between degree of dental fluorosis and its effect on the oral microbial community using a high-throughput sequencing approach. Our results enhances our understanding of how the severity of dental fluorosis influences the oral microbiota and thus provides references for clinical intervention of dental fluorosis and related oral diseases.

## Materials and methods

### Subject recruitment

Informed consent was obtained from all subjects and parents / guardians. All work was approved by the Institutional Ethic Committee of Zunyi Medical University, and all research methods were performed in accordance with relevant guidelines and regulations. A total number of 76 students at Datian Junior High School, Qinglong, Guizhou province, China, an area with endemic coal-fired-pollution-induced dental fluorosis, were diagnosed and classified based on the Dean’s classification system by a dental practitioner (Table 1)^15,16^. Then, all of them were asked to complete a self-report questionnaire. Middle school students ages 12–14 without periodontitis, tooth decay, and tooth loss were included in this study. Individuals subject to antibiotic usage within the last 6 months or professional teeth cleaning within the last 3 months were excluded from the study. To excluding the direct influence of fluoride to oral microbiome, we also excluded the individuals that still exposing to excessive fluoride in the past year. Subjects meeting the above criteria were asked to provide saliva samples into a sterile cup after fasting for over 2 h and rinsing with clear water. The collected saliva was transferred into sterile tubes and placed on ice immediately. All samples were stored at − 80 °C. A total number of 42 students are suitable for sampling criteria (Table 2). We grouped samples into Healthy (Code 0, Dean’s Index), Mild (Code 2 and Code 3), M&S (Moderate/Severe, Code 4 and Code 5), depending on the damage effects of fluoride on teeth surfaces described in the Dean’s index.

**Table 1:** Diagnostic criteria based on Dean’s Index.

Classification	Code	Diagnostic criteria^a^
Normal	Code 0	The surface of the Enamel is smooth and lustrous, usually milky white
Suspicious	Code 1	Enamel transparency has mild change, there are white spots to occasionally see white spots, clinical diagnosis can not be very mild
Very mild	Code 2	The opaque white area on the Enamel surface does not exceed 25% of the tooth surface
Mild	Code 3	The opaque white areas on the enamel surface are extensive, but not more than 50%
Moderate	Code 4	Most of the Enamel Surface Brown Dye, yellow–brown or Brown, teeth have a mild wear, affect the appearance
Severe	Code 5	The Enamel surface is all damaged, the pit defect is obvious, the stain is deep, the tooth Brittleness increases

^a^According to the Enamel Color, luster and the area of the defect to determine the degree of damage, from a person's dentition found the most damaged two teeth score, if two teeth damage degree is different, with the lighter one score.

**Table 2:** Distribution of individuals by sex and Dean’s index.

Groups	Healthy	Mild		M&S^a^	
Dean's index	Code 0	Code 2	Code 3	Code 4	Code 5
Male	6	5	3	6	1
Female	3	3	3	9	3
Total	9	8	6	15	4

^a^M&S represent moderate and severe.

### DNA extraction and sequencing

Total saliva genomic DNA was extracted using the TIANamp Bacteria DNA kit [TIANGEN Biotech (Beijing)] according to the manufacturer’s protocol. The DNA concentration was determined by NanoVue Plus (GE Healthcare, Piscataway, USA). The hypervariable region V4 of bacterial 16S rRNA gene was amplified using KOD-plus-Neo DNA polymerase (Toyobo, Tokyo, Japan) and the universal primers (515F: 5′-GTG YCA GCM GCC GCG GTA A-3′ and 806R: 5′-GTG GAC TAC HVG GGT WTC TAA-3′). Primers were bar coded to allow to multiplex samples. The concentration and purity of PCR products were evaluated using Qubit 2.0 (ThermoFisher) and GE NanoVue (GE Healthcare Biosciences). The library was prepared with TruSeq DNA PCR-Free Sample Prep Kit and sequenced using Illumina HiSeq 2500 sequencer and Hiseq Rapid SBS Kit v2 to generate 2 × 250 bp paired-end reads.

### Sequence analysis

The raw paired-end reads were joined using FLASH^17^ with default parameters. Adaptor sequences of joined reads were removed by cutadapt^18^ (–p-minimum-length 245, –p-overlap 10, –p-no-indels). We detected and removed low quality reads having a low quality 5 bp-window of which the average Q score was less than 30 using the quality filter plugin of QIIME2^19^. A robust denoising method for Illumina data, DEBLUR program^20^ integrated within QIIME2, was used for removal of artificial sequences, error reducing, and obtaining operational taxonomic units (OTUs) at the single-nucleotide level^21^. Naïve Bayesian classifier was trained with Greengenes (13_8 clustered at 99% similarity) database and used for taxonomic annotation of OTUs (confidence threshold: 70%)^22,23^. Further taxonomic annotation at the species level was performed using local BLASTN (ncbi-blast-2.10.0 +)^24^ with the NCBI refseq bacteria 16 s rRNA database (2/11/2020)^25^. In briefly, the representative sequence of each OTUs was aligned with reference sequences of NCBI refseq database. The best hit with > 0.97 of identifies was selected as the taxonomic annotation of species. The number of reads for each sample was subsampled to 6,185 to reduce library size bias before downstream analysis.

### Ecological and statistical analyses

Three measures of alpha diversity [Shannon index, number of observed OTUs, and phylogenetic diversity (Faith's PD)] were calculated using QIIME2. Differences of alpha diversity in different groups based on dental fluorosis levels were tested using Kruskal–Wallis test. Beta diversity was evaluated using Bray–Curtis, Jaccard, weighted Unifrac, and unweighted Unifrac distance to explore the dissimilarity of bacterial communities between groups. The distance matrixes of beta diversity and their corresponding principal coordinate analysis (PCoA) were also calculated in QIIME2. The analyses of similarities (ANOSIM) was conducted to compare the differences in microbiota composition between the groups. R package "ggplot2"^26^ and "pheatmap" were used to comply figures in this study. The differential abundances of bacterial species between Healthy and M&S groups were identified using Linear discriminant analysis Effect Size (LEfSe) analysis^27^, and species with an LDA > 3.0 fold were considered to be significantly different. LEfSe analysis was performed according to Galaxy-based LefSe (http://huttenhower.org/galaxy). The difference in relative abundance of species in different groups were tested using wilcoxon rank sum test, and *P* values < 0.05 were considered significantly different statistically.

## Results

### Summary of sequencing data and alpha diversities

We sequenced the V4 Hypervariable region of 16S rRNA gene to detect bacterial communities from saliva of 42 subjects displaying various levels of dental fluorosis. A total of 2,016,819 pair-end reads with an average of 48,020 per sample ranging from 14,529 to 94,017 were generated using Illumina Hiseq platform. After quality filter and removal of artificial sequences, 1,118,090 high quality reads were obtained, with a mean of 26,621 sequences and a range of 6136–53,444 sequences per sample. According to the DEBLUR program, a total of 981 OTUs (Table S1) were identified by clustering at single-nucleotide level. These OTUs were assigned into 18 bacterial phyla and 185 genera. The total number of sequences of each sample was normalized to the same size of 6136 for downstream analyses.

**Figure 1:**
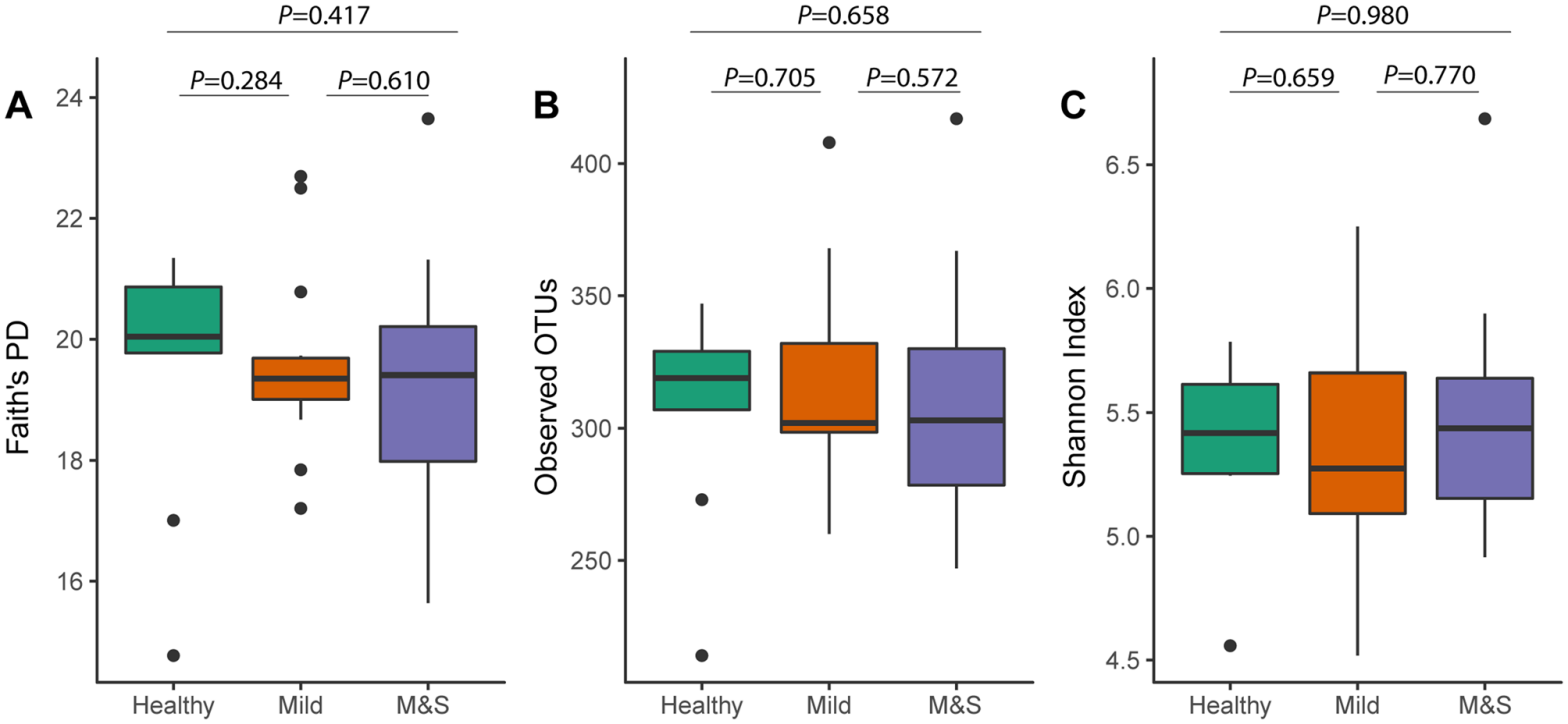
Salivary microbial community alpha diversity box plots showing (**A**) Faith’s PD, (**B**) Observed OTUs, and (**C**) Shannon richness by different degrees of dental fluorosis.

In order to assess the degree effect of dental fluorosis on the alpha diversity of the oral microbiome, we analyzed the species richness (Number of observed OTUs), phylogenetic diversity (Faith’s PD), and species diversity (Shannon index). Compared with Mild and M&S groups, the Healthy group had a higher phylogenetic diversity (Fig. 1A) and species richness (Fig. 1B), although there was no significant statistical difference between all groups (Kruskal–Wallis, Number of Observed OTUs: *P* = 0.811; Faith’s PD: *P* = 0.560) as well as pairwise comparison (Wilcoxon test, *P* > 0.05). The boxplot based on Shannon index (Fig. 1C) which combines the species diversity and evenness shows that the Mild group is lower than the Healthy and M&S groups, but without significant statistical differences.

### Beta diversity shifts in the oral bacterial community of patients with moderate/severe dental fluorosis

**Figure 2:**
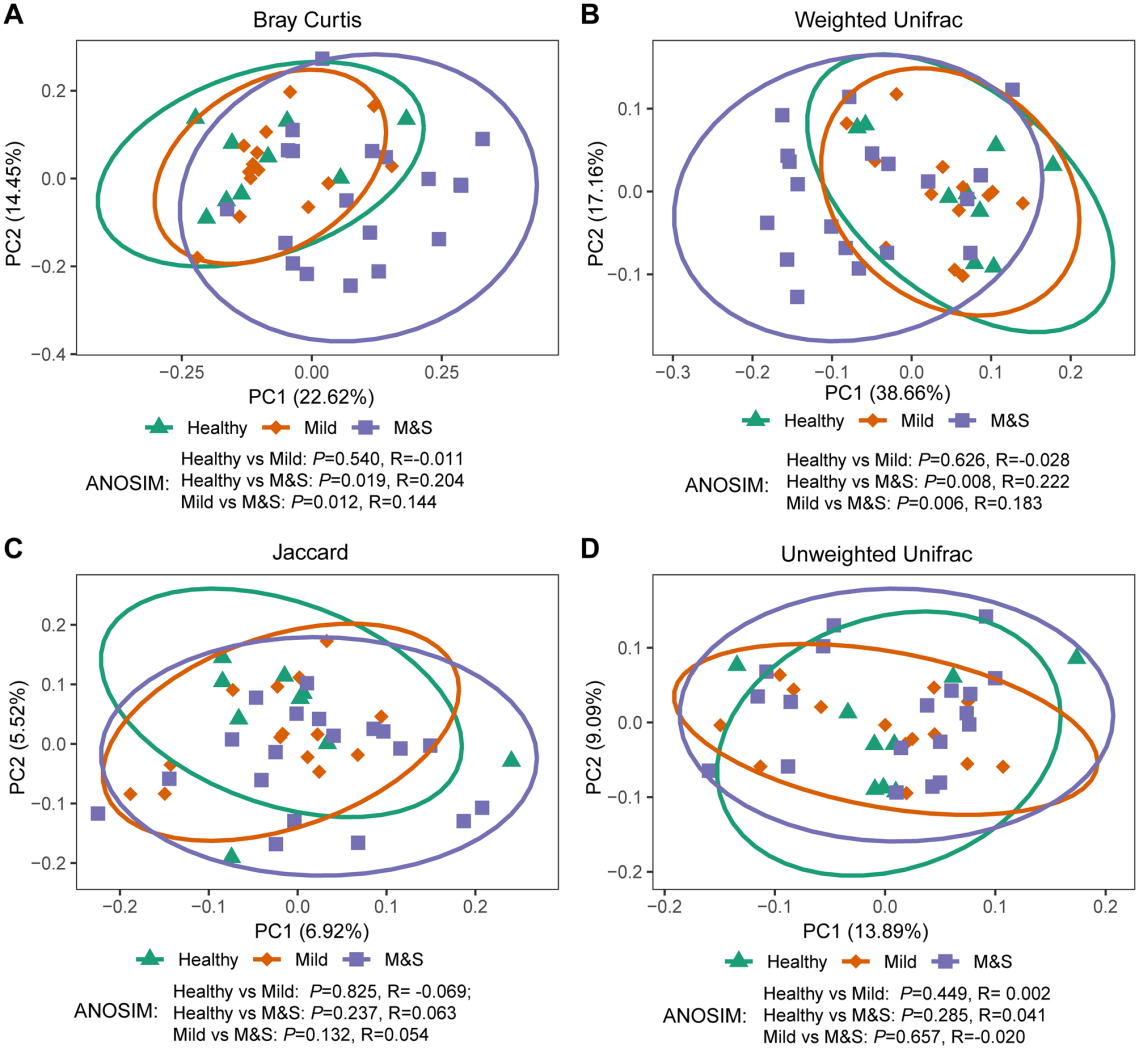
Beta diversity plot of salivary samples based on Principal coordinates analysis (PCoA) of (**A**) Bray–Curtis, (**B**) weighted Unifrac, (**C**) Jaccard, and (**D**) unweighted Unifrac distance. Green/triangle, orange/rhombus, and purple/square represent Healthy, Mild and Moderate/Severe, respectively. The ellipses were calculated and drawn with 0.95 of confidence level using R/ggplot2^26^ (stat_ellipse).

To assess the dissimilarities in community structure and membership of salivary microbiome between groups, beta diversity metrics (Bray Curtis, Jaccard, Weighted Unifrac, and Unweighted Unifrac) were calculated. The PCoA plot based on Bray–Curtis distance (Fig. 2A) shows that the M&S group to be significantly separated from the Healthy (ANOSIM, R = 0.203, *P* = 0.019) and Mild (ANOSIM, R = 0.143, *P* = 0.012) groups. Similarly, we found a significant shift in the M&S group compared with the Healthy (ANOSIM, R = 0.222, *P* = 0.008) and Mild (ANOSIM, R = 0.183, P = 0.006) groups in the PCoA plot based on weighted unifrac distance (Fig. 2B). No significant difference was found between three groups in community membership (Jaccard, Fig. 2C) and unweighted phylogenetic structure (Unweighted Unifrac, Fig. 2D).

### Bacterial taxa significantly changed at the phylum and genus level

**Figure 3:**
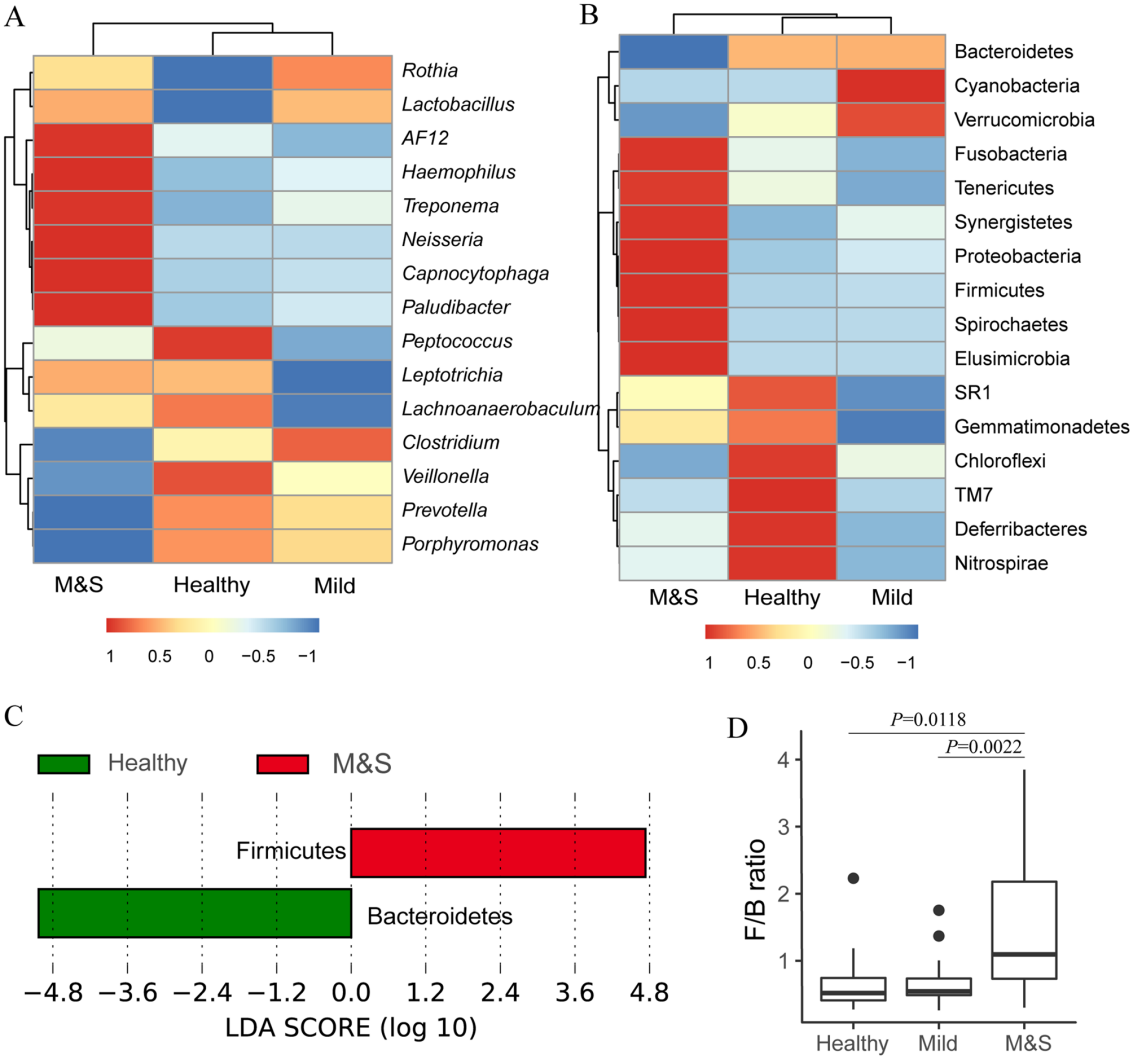
Hierarchically clustered heatmap of taxonomy analysis at the genus (**A**) and phylum (**B**) level using R/pheatmap. (**C**) Significant different taxa at phylum level between Healthy and M&S group based on LEfSe analysis. (**D**) Boxplot for the Firmicutes/Bacteroidetes (F/B) ratio among different groups. The F/B ratio of M&S group was significantly higher than Mild (Wilcoxon Rank Sum Test, *P* = 0.002). There was no statistical difference between Healthy and M&S group (Wilcoxon Rank Sum Test, *P* = 0.012).

At phylum level (Fig. 3A), approximately 94.3% of sequences of all samples were assigned to five major taxonomic groups including Bacteroidetes (35.2%), Firmicutes (27.8%), Proteobacteria (18.2%), Actinobacteria (7.7%), and Fusobacteria (5.4%). Figure 3A shows that the relative abundance of phylum Bacteroidetes decreased in the M&S group compared with the Healthy and Mild groups, while the relative abundance of Firmicutes and Proteobacteria phyla increased. As a result, Bacteriodetes was predominant in the Healthy and Mild groups, while Firmicutes was dominant in the M&S group. LEfSe analysis was applied to identify significant differential taxa between the M&S and Healthy groups at phyla level. Figure 3C shows that phylum Bacteroidetes and Firmicutes were significantly enriched in the Healthy and M&S groups, respectively.

The Firmicutes/Bacteroidetes (F/B) ratio was calculated (Fig. 3D). The F/B ratio of M&S (Average = 1.39) was significant greater than Mild (Average = 0.68, Wilcoxon Rank Sum Test, *P* = 0.0022) and Healthy (Average = 0.73, Wilcoxon Rank Sum Test, *P* = 0.0118).

At the genus level (Fig. 3B), the most abundant taxonomy was *Prevotella* (16.0%), followed by *Porphyromonas* (12.7%), *Streptococcus* (10.9%), and *Neisseria* (8.8%), constituting 48.4% of the overall abundance. A change trend of dominant bacterial genera was observed. This trend revealed the relative proportion of both *Prevotella* and *Porphyromonas,* belonging to phylum Bacteroidetes, decreased in the M&S group compared with the Heathy and Mild groups. The relative proportion of *Streptococcus,* belonging to phylum Firmicutes, increased which could be the primary reason for the change of Firmicutes/Bacteroidetes ratio in the M&S group.

### Differentially represented species in M&S and healthy groups

**Figure 4:**
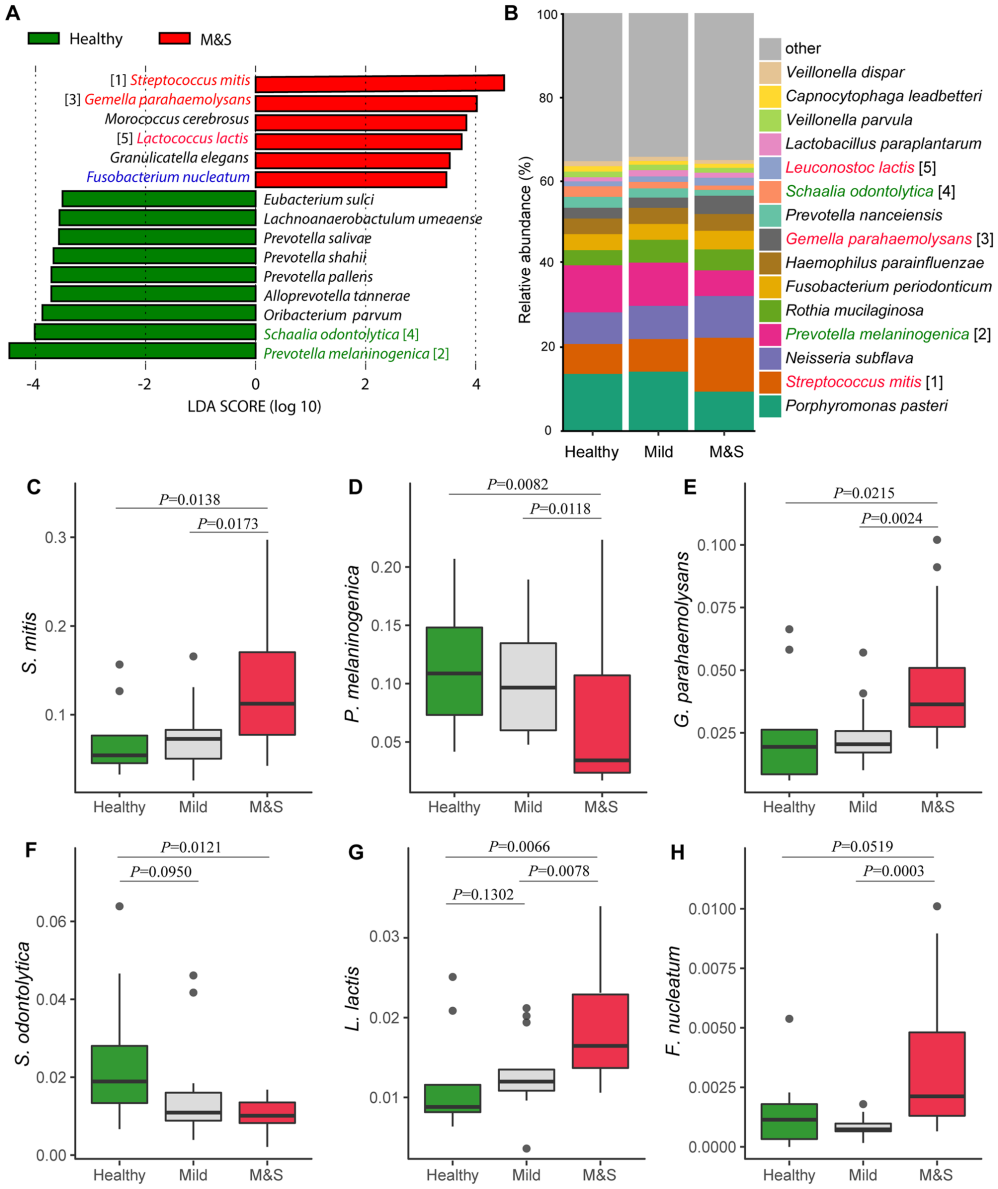
(**A**) Significantly different bacterial species between Healthy and M&S group based on LEfSe analysis. The number in square brackets represents the species also present as top 15 abundant bacterial species and corresponds to the number of plot B. (**B**) Average relative abundance of the top15 most abundant bacterial species. Boxplot for the relative abundance of *Streptococcus mitis* (**C**)*, Prevotella melaninogenica* (**D**)*, Gemella parahaemolysans* (**E**)*, Schaalia odontolytica* (**F**)*, Lactococcus lactis* (**G**)*, and Fusobacterium nucleatum* (**H**). The difference of relative abundance of species in different groups were tested using wilcoxon rank sum test.

Based on our results, the oral microbiome of patients with moderate or severe dental fluorosis was largely altered. In order to further explore the changed microbial taxa in the M&S group, we identified significant bacterial differences between M&S and Healthy using LEfSe analysis at species level. *Streptococcus mitis*, *Gemella parahaemolysans*, *Morococcus cerebrosus*, *Lactococcus lactis*, *Granulicatella elegans*, *Fusobacterium canifelinum*, *Fusobacterium nucleatum*, and *Lactobacillus delbrueckii* were significantly enriched in the M&S group, while *Prevotella melaninogenica*, *Schaalia odontolytica*, *Prevotella pallens*, *Leptotrichia shahii*, *Prevotella salivae*, and *Bifidobacterium dentium* were mostly associated with the Healthy group (Fig. 4A). Interestingly, five of these species (Fig. 4C–G) were listed in the top 15 abundant species (Fig. 4B, Table S2). Except for *Schaalia odontolytica* (Fig. 4F), the other four species including *Streptococcus mitis* (Fig. 4C), *Prevotella melaninogenica* (Fig. 4D), *Gemella parahaemolysans* (Fig. 4E), and *Lactococcus lactis* (Fig. 4G) each had a closer relative abundance between the Mild and Healthy groups compared to the M&S group (wilcoxon rank sum test, *P* < 0.05). In addition, *Fusobacterium nucleatum* (Fig. 4H), which was associated with digestive tract cancers^28,29^, had a higher relative abundance in M&S group than Mild and Healthy groups. The statistical difference was confirmed only between M&S and Mild (Wilcoxon Rank Sum Test, *P* = 0.0003), but not between M&S and Healthy (Wilcoxon Rank Sum Test, *P* = 0.012).

## Discussion

The detrimental effects of dental fluorosis on the appearance and quality of dental enamel is clearly known. However, there is limited research explaining the effects of dental fluorosis on the oral microbiome. In this study, we characterized the oral microbial community structure of salivary samples using high-throughput sequencing technology. The data reveals that the oral microbial community structure is influenced by moderate and severe dental fluorosis but not by the mild dental fluorosis.

Beta-diversity metrics reflect the taxonomic similarity between different samples. In this study, differences between M&S group and the other two (Healthy and Mild) were found in PCoA plot based on Bray–curtis and weighted unifrac dissimilarity, but not in Jaccard and unweighted unifrac. Metrics of beta diversity with different content information can be responsible for this inconsistency. The Jaccard metric explains microbial taxonomic variation (presence or absence) among samples (qualitative data), in contrast the Bray–Curtis metric provide additional abundance information (quantitative data). Similarly, weighted Unifrac considers both phylogeny and abundance of microbiota, while unweighted Unifrac doesn’t consider abundant information. It suggested that moderate/severe dental fluorosis may affect the relative abundance of bacterial species, but not variety. The phyla Firmicutes and Bacteroidetes have critical roles in the human gut. Previous studies suggested that the F/B ratio was an important indicator of the gut microbiota and its relationship with age, body weight, food, metabolism, and disease^30–32^. Similarly, F/B ratio of the oral microbiome was also analyzed in this study. Interestingly, F/B ratio of M&S group was higher than both the Healthy and Mild groups. Based on our results, there was no evidence displaying the F/B ratio relationship between the oral microbiome and oral health status. However, the results indicated that moderate and severe dental fluorosis may cause oral microbial shift.

In this study, several important bacterial taxa of the M&S group were significantly different compared to both Healthy and Mild groups, while Healthy and Mild groups shared a similar bacterial community structure. Engström’s study^33^ suggested that fluoridated milk did not alter the composition of the salivary bacterial community structure. Indeed, fluoride mouthwash has little impact on the oral microbiome^34^. Study participants, with or without dental fluorosis, did not expose to excessive fluoride for at least 1 year. Therefore, we considered the possibility that fluoride caused the changes in the oral microbiome. The Dean’s Index for dental fluorosis is a widely used classification system that estimates the severity of fluorosis based on clinical appearance of the teeth. In this system, moderate and severe degrees of fluorosis is based on the amount of damage to the enamel surface while very mild and mild is described by the occurrence of white opaque areas. We inferred that surface deterioration caused by moderate and severe dental fluorosis made it difficult for patients to clean food and plaque from their teeth, thereby exacerbating poor oral hygiene. In Michel-Crosato’s survey, oral hygiene was the most daily activity affected by dental fluorosis^35^. Vora’s study^4^ provided the evidence that dental fluorosis intensified problems with oral hygiene. Therefore, we concluded that excess fluoride as the primary factor for weakening tooth surface enamel leading to poor oral hygiene and, in turn, causing the alteration of the oral microbiota. However, more investigations are needed to understand more thoroughly the influence moderate and severe dental fluorosis has on oral hygiene and the oral microbiota.

Dental fluorosis is strongly associated with dental caries^36^. It is commonly known that dental caries are caused by the presence of acid produced by certain bacteria^37^. In this study, two acid forming bacterial species, *Streptococcus mitis* (*S. mitis*) and *Lactococcus lactis* (*L. lactis*), were dominant microbiota members of the M&S group. *S. mitis* was listed as the second most abundant species, and is usually considered a common commensal species of the human mouth however, it is also known as an opportunistic pathogen^38^. According to previous research, *Streptococcus* species have the ability to produce acidic end-products via the fermentation of carbohydrates leading to excessive acidification of the oral environment which is directly linked to the formation of dental caries^39^. The acid-producing ability of *S. mitis* is at its maximum at PH 7.0 and 6.0 in the oral environment^40^. Thus, *S. mitis* is directly associated with dental caries formation^41,42^. *L. lactis* is a common industrial fermentation bacteria, and is a known member of the oral cavity where it ferments sugars into lactic acid. There appears to be no evidence that demonstrates *L. lactis* is strongly associated with dental caries. However, increased acid-producing ability of oral bacteria indicates that glycolysis is active in the oral cavity, which increases the risk of dental caries occurrence and progression.

*Fusobacterium nucleatum* (*F. nucleatum*) is not only a resident bacteria in the oral cavity but also an opportunistic pathogen that can cause periodontal disease. It is also linked to gastrointestinal^29,43^, esophageal^28^, and oral cancers^44^. Yamamura^28^ detected significantly higher abundance of *F. nucleatum* in esophageal cancer tissues than the corresponding normal esophageal mucosa. It was also discovered to be associated with a particular tumor stages. Most recently, Menya^6^ suggested that moderate/severe dental fluorosis was strongly associated with a 9.4-fold increased risk of esophageal squamous cell carcinoma (ESCC) compared with a no dental fluorosis population in Africa. In this study, *F. nucleatum* was significantly enriched in the M&S group and significantly higher than the Mild and Healthy groups. This suggests of a potential role that *F. nucleatum* plays in causing ESCC in individuals with moderate/severe dental fluorosis.

## Conclusion

In conclusion, our study provided the first evidence that the oral bacterial community was significantly changed in moderate and severe dental fluorosis, compared with individuals with mild dental fluorosis and those with healthy teeth. Using LEfSe analysis, we identified several bacterial species enriched in moderate and severe fluorosis. Future studies are desired to explore the roles of these bacteria in fluorosis development. In addition, the roles of these bacteria in oral health and related diseases warrant more consideration in patients with moderate and severe fluorosis.

## Supplementary Information

Supplementary Information
