# Supplementary material for: Structural changes in the oral microbiome of the adolescent patients with moderate or severe dental fluorosis
Source: Sci Rep. 2021 Feb 3;11:2897. doi: 10.1038/s41598-021-82709-z (PMC7859183; doi:10.1038/s41598-021-82709-z)
Supplement: Supplementary file 1 — Supplementary Information [file 41598_2021_82709_MOESM1_ESM.docx]

Supplementary Information

**Structural changes in the oral microbiome of the adolescent patients with moderate or severe dental fluorosis**

Qian Wang^1, 2^, Xuelan Chen^1, 4^, Huan Hu^2^, Xiaoyuan Wei^3^, Xiaofan Wang^3^, Zehui Peng^4^, Rui Ma^4^, Qian Zhao^4^, Jiangchao Zhao^3, *^, Jianguo Liu^1, *^, Feilong Deng^1, 2, 3, *^

^1^ Special Key Laboratory of Oral Disease Research, Research Center for Medicine and Biology, Zunyi Medical University, Zunyi, China; ^2^ Special Key Laboratory of Microbial Resources and Drug Development, Research Center for Medicine and Biology, Zunyi Medical University, Zunyi, China; ^3^ Department of Animal Science, Division of Agriculture, University of Arkansas, Fayetteville, AR, USA; ^4^  School of Stomatology, Zunyi Medical University, Zunyi, China

Table S1. Percentage of OTUs relative abundance in each subject

<https://doi.org/10.6084/m9.figshare.13185452.v1>

Table S2. Species relative abundances of each subject at species level

<https://doi.org/10.6084/m9.figshare.13185449.v1>
